# Assessment and optimization of *Theileria parva* sporozoite full-length p67 antigen expression in mammalian cells

**DOI:** 10.1371/journal.pntd.0005803

**Published:** 2017-08-11

**Authors:** Giulia Tebaldi, Laura B. Williams, Andrea E. Verna, Francesca Macchi, Valentina Franceschi, Lindsay M. Fry, Donald P. Knowles, Gaetano Donofrio

**Affiliations:** 1 Department of Medical-Veterinary Science, University of Parma, Parma, Italy; 2 United States Department of Agriculture and Department of Veterinary Microbiology & Pathology, Animal Disease Research Unit, Agricultural Research Service, Washington State University, Pullman, WA, United States of America; 3 Paul G. Allen School for Global Animal Health, Washington State University College of Veterinary Medicine, Pullman, WA, United States of America; University of Iowa, UNITED STATES

## Abstract

Delivery of various forms of recombinant *Theileria parva* sporozoite antigen (p67) has been shown to elicit antibody responses in cattle capable of providing protection against East Coast fever, the clinical disease caused by *T*. *parva*. Previous formulations of full-length and shorter recombinant versions of p67 derived from bacteria, insect, and mammalian cell systems are expressed in non-native and highly unstable forms. The stable expression of full-length recombinant p67 in mammalian cells has never been described and has remained especially elusive. In this study, p67 was expressed in human-derived cells as a full-length, membrane-linked protein and as a secreted form by omission of the putative transmembrane domain. The recombinant protein expressed in this system yielded primarily two products based on Western immunoblot analysis, including one at the expected size of 67 kDa, and one with a higher than expected molecular weight. Through treatment with PNGase F, our data indicate that the larger product of this mammalian cell-expressed recombinant p67 cannot be attributed to glycosylation. By increasing the denaturing conditions, we determined that the larger sized mammalian cell-expressed recombinant p67 product is likely a dimeric aggregate of the protein. Both forms of this recombinant p67 reacted with a monoclonal antibody to the p67 molecule, which reacts with the native sporozoite. Additionally, through this work we developed multiple mammalian cell lines, including both human and bovine-derived cell lines, transduced by a lentiviral vector, that are constitutively able to express a stable, secreted form of p67 for use in immunization, diagnostics, or *in vitro* assays. The recombinant p67 developed in this system is immunogenic in goats and cattle based on ELISA and flow cytometric analysis. The development of a mammalian cell system that expresses full-length p67 in a stable form as described here is expected to optimize p67-based immunization.

## Author summary

East Coast fever, caused by the tick-borne protozoan parasite *Theileria parva*, is a disease that results in significant bovine morbidity, mortality, and production losses in regions of sub-Saharan Africa. Susceptible cattle develop clinical signs within a 7–14 days of exposure, which often progress to severe pulmonary edema and death. Control of East Coast fever in affected regions of Africa is largely prohibited by the lack of an affordable and efficacious vaccine. Furthermore, pastoralist farmers in affected regions of Africa often lack resources to prevent losses due to East Coast fever, so these production losses play a significant role in food security and protein availability. Experimental immunization of cattle with a recombinant *T*. *parva*-derived antigen, p67, has shown promise in preventing East Coast fever, but this antigen is extremely difficult to produce in full-length in sufficient quantities, and results of immunization studies using truncated recombinant p67 products are highly inconsistent. In this study, p67 antigen production was optimized and produced for use in future immunization studies. Optimization of p67-based immunization strategies is an important step forward in the development of a sustainable, next-generation vaccine against *T*. *parva*, which is urgently needed to minimize losses associated with East Coast fever.

## Introduction

*Theileria parva (T*. *parva)* is an intracellular protozoan belonging to the *Theileria* genus, order Piroplasmida and phylum Apicomplexa [1–3]. This parasite is the causative agent of East Cost fever (ECF), an acute and often lethal disease affecting cattle in different countries of eastern, central and south Africa [4]. *T*. *parva* is transmitted to cattle by *Rhipicephalus appendiculatus* ticks. Once within cattle, infectious sporozoites enter B and T lymphocytes and mature into schizonts [5,6]. Schizonts primarily induce T-cell transformation and proliferation [7–9], which is pharmacologically reversible using anti-*Theileria* drugs [9–11]. *T*. *parva* infection often results in pulmonary edema and death [12]. ECF is a leading cause of death in cattle in sub-Saharan Africa, resulting in approximately US$ 168 million in annual economic losses and death of over 1.1 million cattle [4].

The disease was conventionally controlled by acaricide use and chemotherapy. However, the rapid development of acaricide-resistance in tick populations and the high cost of veterinary care required for timely administration of chemotherapy limits the control of ECF. A mode of *T*. *parva* prevention is the infection and treatment method (ITM). ITM involves infection of cattle with live, *T*. *parva* sporozoites and concurrent treatment with a long-acting form of oxytetracycline. Although effective, production of ITM tabulates is extremely costly and inefficient, and the requirement of co-treatment with oxytetracycline makes this form of prevention too costly for many pastoralist farmers. Thus, new, safer and more economically sustainable methods of prevention, such as a next-generation vaccine, are urgently needed. [4,13].

During the last 20 years a strong endeavor has been made, with variable results, to search for an alternative vaccine to prevent ECF [13,14]. The majority of the work focused on the isolation and delivery of defined *T*. *parva* sporozoite and schizont antigens. The most protective *T*. *parva* sporozoite antigen identified to date is the surface protein, p67 [15]. p67 is recognized by neutralizing antibodies detected in immunized animals with *T*. *parva* sporozoites. Moreover, immunized mice with *T*. *parva* sporozoites produced neutralizing monoclonal antibodies and most of these antibodies recognized p67 on the sporozoites surface [15–17].p67 is essential for host cell recognition and sporozoite entry, and its expression is strictly limited to the sporozoite stage while the kinete, schizont, merozoite, and piroplasm stages of the parasite do not express p67 [18].

Several studies have been carried out using recombinant p67 expressed by different systems, administered by different adjuvants, and delivered by a variety of vectors [4,13]. Paradoxically, better results have been obtained using adjuvanted p67 protein expressed in *E*. *coli* or insect cells, rather than vector-delivered p67 [4,13,19]. This could be attributed to the low level of p67 stable form expression in mammalian cells. Although some papers reported the use of recombinant viruses to deliver the p67 ORF, these studies provided no data regarding the efficiency of p67 expression after cell transduction [19]. Vector-based delivery, and especially viral vector-based heterologous antigen delivery, needs careful regard considering that the immune system has evolved a sophisticated mechanisms array to both detect and eliminate invading viruses. Viral vectors also deliver the ORF antigen directly into the host cell, potentially conferring a high-level expression of the ORF antigen. Hence, expression cassette optimization represents a crucial step for a successful vector antigen construction.

In the present work, full-length p67 protein expression in mammalian cells has been achieved and optimized for the first time, paving the way for further p67 vectorialization for immunization studies and ECF vaccine development.

## Materials and methods

### Cell lines

Bovine Bone Marrow Stromal Cells cell (BBMC), Goat Skin Stromal cells (GSSC), Swine Adipose Derived Stromal cells (SADSC), Equine Adipose Derived Stromal cells (EADSC) and Alpaca Skin Stromal cells (ASSC) were derived, immortalized and maintained as described in [20], [21], [22], [23] and [24]. HEK (Human Embryo Kidney) 293T (ATCC: CRL-11268), BBMC, GSSC, SADSC, EADSC and ASSC were cultured in Eagle's Minimal Essential Medium (EMEM, Gibco) containing 10% fetal bovine serum (FBS), 2 mM of L-glutamine (Gibco), 100 IU/mL of penicillin (Gibco), 100 μg/mL of streptomycin (SIGMA) and 0.25 μg/mL of amphotericin B (Gibco) and were incubated at 37°C, 5% CO_2_ in a humidified incubator.

### Constructs generation

The synthetic *T*. *parva* p67 ORF was excised from pEX-K4p67 (Eurofins, Genomics) via cutting with NheI and HindIII restriction enzymes. The 2246bp p67 fragment was then cloned into NheI/HindIII cut pEGFP-C1 shuttle vector (Clontech) to generate pCMV-p67.

The p67 secreted fragment (pCMV-p67ΔTM), without the trans-membrane domain, was obtained by PCR amplification from pCMV-p67 using NheI p67 sense (5’-cgtcagatccgctagcccaccatgcagatcacccagttcc -3’) and 685-SalI p67 antisense (5’-cccgtcgaccttcttcttcagcttctggatc-3’) primers. The PCR amplification reaction was carried out in a final volume of 50 μL, containing 1X Pfu buffer (20 mM Tris–hydrochloride pH 8.8, 10 mM (NH_4_)_2_SO_4_, 10 mM KCl, 100 ng/mL BSA, 0.1% TritonX-100, 2 mM MgSO₄, 10% Dimethyl Sulfoxide (DMSO)), 0.2 mM deoxynucleotide triphosphates, and 0.25 μM of each primer. One hundred nanograms of DNA were amplified over 35 cycles, each cycle consisting of 1 min of denaturation at 94°C, 1 min of primer annealing at 60°C and 2.30 min of chain elongation with 1U of Pfu DNA polymerase (Fermentas) at 72°C. The generated 2139bp p67ΔTM fragment was checked in 1% agarose gel and visualized after ethidium bromide staining in 1X TAE buffer (40 mM Tris-acetate, 1 mM EDTA). The amplified fragment was cut with NheI/SalI, ligated in NheI/SalI digested GFP (green fluorescent protein) ORF emptied pEGFP-C1 in order to obtain pCMV-p67ΔTM. p67 mutated protein, with the seven putative arginine glycosylation sites substituted with glutamine, was NheI/SmaI cut out from pEX-k4ΔGlyco (Eurofins, Genomics) and the 2152bp fragment was cloned into NheI/SmaI cut pINT2-EGFP [25] shuttle vector in order to obtain pCMV-p67ΔGlyco. A lentiviral transfer vector, pEF1α-p67ΔTM-iresGFP, delivering the p67 secreted form was obtained through ligation of the expression cassette, excised from blunt ended NheI/BamHI cut pCMV-p67ΔTM, into PmeI cut pWPI (addgene).

### Lentivirus reconstitution and transduction

Briefly, HEK 293T cells were transfected in a T175 cm^2^ flask with 25 μg of transfer vector pEF1α-p67ΔTM-iresGFP, 13 μg of packaging vector p8.74, 10 μg of pseudotyping vector pMD2 and 10 μg of pREV using Polyethylenimine (PEI) transfection reagent (Polysciences, Inc.). Briefly, 58 μg of DNA were mixed with 116 μg of PEI (1mg/mL) (ratio 1:2 DNA-PEI) in 3 mL of Dulbecco’s modified essential medium (DMEM) high glucose (Euroclone) without serum. After 15 min at room temperature (RT), 14 mL of medium without serum were added and the transfection solution was transferred to the cells (monolayer) and left for 6 hours at 37°C with 5% CO_2_, in a humidified incubator. The transfection mixture was then replaced with 25 mL of fresh medium EMEM, with 10% FBS, 100 IU/mL of penicillin, 100 μg/mL of streptomycin and 0.25 μg/mL of amphotericin B and incubated for 24 hours at 37°C with 5% CO_2_. 48 hours after transfection, the flask was stored at -80°C and the lentivirus was obtained by freezing and thawing cells three times. Subsequently, the supernatant was first clarified at 3500rpm for 5 min at 4°C, filtered through a 0.45 μm filter (Millipore) and stored at -80°C. To obtain stably transduced HEK-p67ΔTM and BBMC-p67ΔTM cell lines, 1x10^5^ cells were infected with 2x10^5^ TU (transducing units) of viral reconstituted pEF1α-p67ΔTM-iresGFP. Twenty-four hours later, the culture medium was replaced with fresh medium supplemented with 10% of FBS and the cells were observed via fluorescence microscopy to monitor the transduction. GSSC, SADSC, EADSC and ASSC were similarly transduced.

### Transient transfection

HEK 293T cells were seeded into six-well plates (3x10^5^ cells/well) and incubated at 37°C with 5% CO_2_. When cells were sub-confluent, the culture medium was removed and the cells were transfected with pCMV-p67, pCMV-p67ΔTM, pCMV-p67ΔGlyco, psecE2 [26,27] and pEGFP-C1 using PEI transfection reagent (Polysciences, Inc.). Briefly, 3 μg of DNA were mixed with 7.5 μg PEI (1mg/mL) (ratio 1:2.5 DNA-PEI) in 200 μL of Dulbecco’s modified essential medium (DMEM) high glucose (Euroclone) without serum. After 15 min at RT, 800 μL of medium without FBS were added and the transfection solution was transferred to the cells (monolayer) and left for 6 hours at 37°C with 5% CO_2_, in a humidified incubator. The transfection mixture was then replaced with fresh medium EMEM, with 10% FBS, 100 IU/mL of penicillin, 100 μg/mL of streptomycin and 0.25 μg/mL of amphotericin B and incubated for 24 hours at 37°C with 5% CO_2_. To test the supernatant protein expression, the transfection mixture was replaced with fresh medium DMEM/F12 (ratio 1:1) without serum and incubated for 48 hours at 37°C with 5% CO_2_.

### Western immunoblotting

Protein cell extracts were obtained from T25cm^2^ confluent flasks of transfected HEK 293T, HEK-p67ΔTM and BBMC-p67ΔTM by adding 100 μL of cell extraction buffer (50 mM Tris-HCl, 150 mM NaCl, and 1% NP-40; pH 8). Cell extracts were electrophoresed through 10% sodium dodecyl sulfate-polyacrylamide gel electrophoresis (SDS-PAGE). Different SDS-PAGE loading buffer denaturing conditions were also used to evaluate a possible p67 aggregation status: SDS concentration (0 to 5%) and heat treatment length (24 hours at 80°C). After protein transfer onto nylon membranes by electroblotting, membranes were incubated with Anti-p67 monoclonal antibody, AR22.7 [28], diluted 1:5.000 and then with a secondary antibody probed with horse radish peroxidase-labelled Anti-Mouse immunoglobulin (Sigma), diluted 1:10.000 to be visualized by enhanced chemiluminescence (ECL Kit, Pierce). Also cell supernatants, obtained from HEK 293T transfected with pCMV-p67ΔTM and HEK-p67ΔTM or BBMC-p67ΔTM, were collected at different time points (4, 8, 24 and 48 hours after serum free medium DMEM-F12 secretion condition) and analyzed through 10% SDS–PAGE. Protein loading was assessed by Commassie Brilliant Blue staining of the membrane as previously described [29].

### Animals and ethics statement

One adult goat and two cattle were used for the *in vivo* immunization study. Animal Use Protocol 04596, entitled “Development of Bovine Herpesvirus-4 as a Vaccine Vector for *Theileria parva* in Cattle" was approved by the Washington State University Institutional Animal Care and Use Committee (IACUC) on 11/17/2014. Washington State University is a USDA registered research facility (43-R-011), is regularly inspected and files all required documentation, including an annual report. In addition, under the provisions of the Public Health Service Policy on the Humane Care and Use of Laboratory Animals, the University files required assurance documents to the Office of Laboratory Animal Welfare (OLAW). (OLAW Assurance Number A-3225-01, effective from March 4, 2013 through March 31, 2017). The Animal Care and Use Program at Washington State University is fully accredited by the Association for Assessment and Accreditation of Laboratory Animal Care, International (AAALACI), continuing accreditation notification July 18, 2012.

After pre-immune blood sample collection, both the goat and the cattle were immunized intramuscularly with 2 mL of p67ΔTM clarified (~14 μg) supernatant in addition to 2 mL of Squalene-based oil-in-water adjuvant (AddaVax InvivoGen). All animals were boosted 21 days after the first immunization with the same procedure. The last blood samples were collected 42 days after p67ΔTM supernatant immunization.

### Sample collection and ELISA procedure

Serum samples were drawn before immunization and at scheduled times and processed for ELISA assays. Briefly, microplates (Microlon High Binding) were coated overnight at 4°C with 50μL/well p67ΔTM protein clarified supernatant obtained from T175 cm^2^ of pEF1α-p67ΔTM-iresGFP lentiviral vector stably transduced HEK 293T cells and diluted in 0.1 M carbonate/bicarbonate buffer pH 9.6. After blocking with 1% bovine serum albumin (BSA), 1:100 diluted serum samples were incubated for 1 hour at room temperature. After 3 washing steps in PBS+Tween 0.05%, 50 μL of diluted 1:20.000 rabbit Anti-Bovine immunoglobulin G-HRP (Sigma) was added to each well and the plate was incubated as above. Following the final washing step, the reaction was developed with 3,3′,5,5′-tetramethylbenzidine (TMB, IDEXX), stopped with 0.2 M H_2_SO_4_ and read at 450 nm.

### Flow cytometry assay

To evaluate the presence of p67 protein on the cell surface and the presence of specific antibodies in the sera of immunized animals, a flow cytometry assay was performed on pCMV-p67 transfected HEK 293T cells expressing p67. The cells plated in a T75 cm^2^ flask were transfected with 22.5 μg of pCMV-p67 DNA, 67.5 μg of PEI (ratio 1:3 DNA-PEI) in 1.5 mL of Dulbecco’s modified essential medium (DMEM) high glucose (Euroclone) without serum. After 15 min at RT, 6 mL of medium without serum were added and the transfection solution was transferred to the cells (monolayer) and left for six hours at 37°C with 5% CO_2_, in a humidified incubator. After six hours, the transfection mixture was replaced with fresh EMEM with 10% FBS. The following day, the transfected cells were washed with sterile PBS to remove any traces of serum and subsequently detached with a PBS-EDTA solution (50 μL of EDTA Ethylenedinitrilotetraacetic acid 0.5 M in a final volume of 50mL).

2x10^5^ re-suspended cells, for every sample (a total of 6 samples plus a control represented by cells only) were centrifuged at 1200 rpm for 4 min at RT. The pelleted cells were incubated at RT for 20 min with inactivated sera diluted 1:20 in 1mL PBS-FBS 2% final volume solution. Next, cells were centrifuged at 1200 rpm for 4 min at RT, washed with 1mL of PBS-FBS 2% solution, re-centrifuged as before to remove the wash buffer, and incubated with secondary Donkey Anti-Goat Fitc antibody (donkey Anti-Goat IgG-FITC: sc-2024 Santa Cruz Biotechnology, inc.) 1:200 diluted in a final volume of 200μL of PBS-FBS 2% and secondary Anti-Bovine IgG (whole molecule)–FITC antibody produced in rabbit (Sigma-Aldrich) 1:200 diluted in a final volume of 200μL of PBS-FBS 2% for goat and cattle sera respectively. As a negative control, the pre-immune sera were employed at the same dilution.

### PNGase F digestion

PNGase F was purchased from NEW ENGLAND BioLabs and tested as suggested by the company user manual. HEK-p67ΔTM and psecE2 transfected HEK cells serum free medium clarified supernatants, collected after 48 hours of secretion, were digested with PNGase F that cleaves between the innermost GlcNAc and asparagine residues (of high mannose, hybrid, and complex oligosaccharides) from N-linked glycoproteins. To optimize the electrophoretic migration according to the acrylamide gradient (4–15%), the supernatant samples were analyzed through BIO-RAD Criterion™ TGX™ (Tris-Glycine eXtended) precast gels for SDS-PAGE. p67 detection was performed by Western immunoblotting as described above.

## Results

### Synthetic gene design and codon usage adaptation

Before attempting the generation of a suitable expression cassette for the *T*. *parva* p67 ORF gene in mammalian cells, the nucleotide composition of p67 gene was taken into account to prevent poor gene expression due to differences in codon usage between apicomplexan and mammalians cells. p67 codon usage was first adapted to the human genome codon usage using the Jcat codon adaptation tool (http://www.jcat.de/)). In general, the *T parva* genome has a low GC content of 34.1%, [30], and specifically, the p67 ORF has a GC content of 43% **(S1A and S1C Fig)**. Adaptation to the human genome codon usage shifted the GC content from 43% to 68% **(S1B and S1D Fig)**. Starting from the p67 human codon usage adapted ORF, a Kozak’s sequence (to improve the translation) and two restriction enzyme sites (to facilitate the sub-cloning in a suitable vector) were added at the ends of the ORF.

### Topological prediction, rational construct design and transient expression of p67 in a membrane linked form

**Fig 1:**
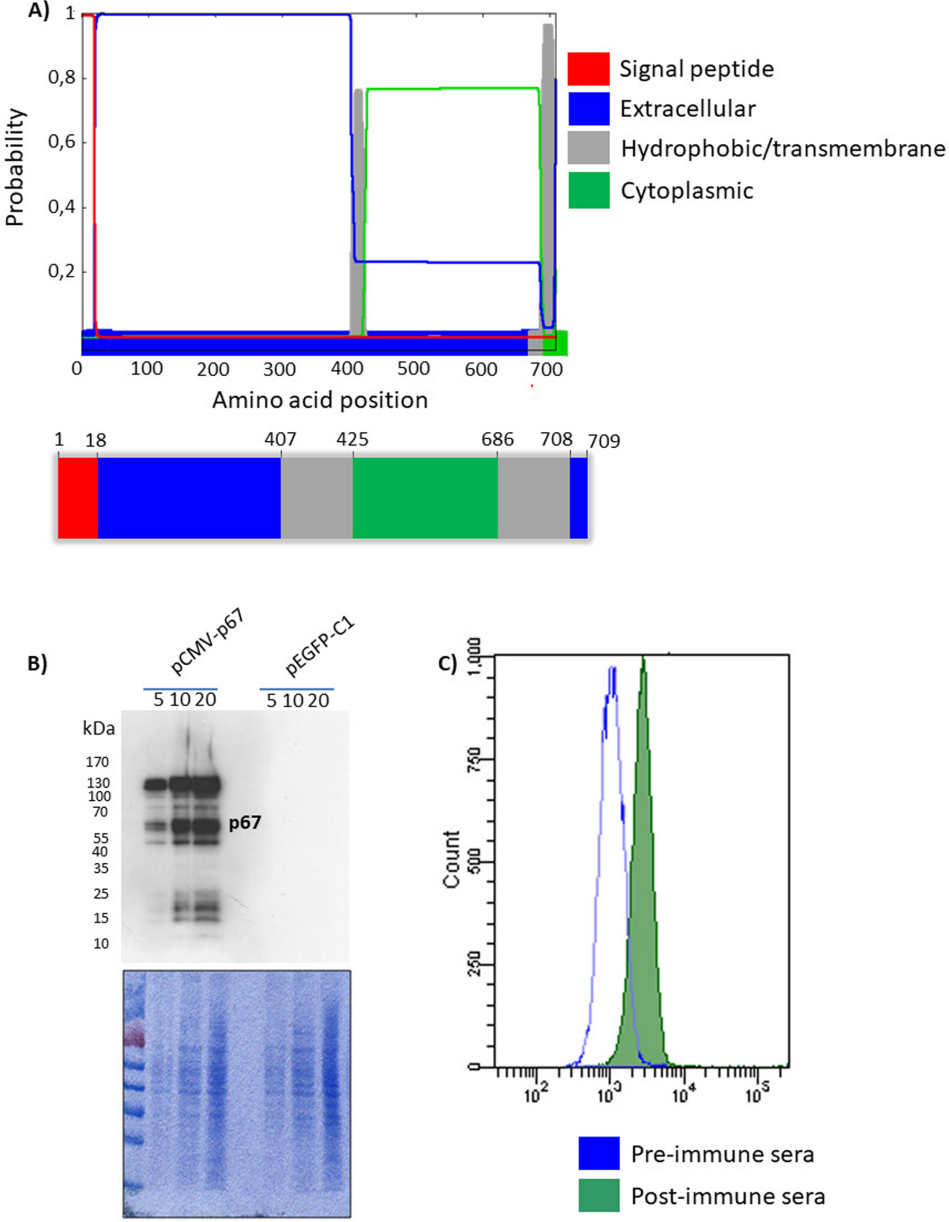
p67 expression by mammalian cells. **A)** Server output prediction of transmembrane topology and signal peptides from the amino acid sequence of p67 protein, along with a cartoon (not on scale) of the p67 predicted protein domains. **B)** Western immunoblotting of pCMV-p67 (membrane linked form) transfected HEK 293T cells extracts. Lanes were loaded with different amounts of total protein cell extract (5, 10 and 20 μg); cells transfected with pEGFPC-1 served as negative controls (*Mock*). Protein loading is shown by membrane staining by Commassie Brilliant Blue. **C)** Flow cytometry analysis of p67 expression on the cell surface of pCMV-p67 transfected cells. The green peak corresponds to the post-immune serum from a p67 immunized goat and the blue peak corresponds to the pre-immune sera which was used as a control.

According to its amino acid sequence and as predicted by Phobius (http://phobius.sbc.su.se/) **(Fig 1A),** a server for prediction of transmembrane domains and signal peptides, and in agreement with a previously published paper [31], *T*. *parva* p67 seems to have an amino-terminal signal peptide (from aa1 to aa18), an extracellular domain (from aa19 to aa406) a hydrophobic region (from aa407 to aa425), a cytoplasmic domain (from aa426 to aa685) a transmembrane domain (from aa686 to aa708) and an extracellular single amino acid (aa709). Therefore, based on this prediction, p67 should be expressed in a eukaryotic expression vector as a full length membrane-linked protein. The synthetic p67 ORF was placed under transcriptional control of the CMV promoter and the bovine growth hormone polyadenylation signal to obtain the pCMV-p67 construct. Transiently pCMV-p67 transfected HEK 293T cells expressed p67, as shown by Western blotting **(Fig 1B)**, which was displayed on the cell surface as shown by flow-cytometry **(Fig 1C)** using an immunized goat serum.

### p67 expressed in mammalian cells is a Type I integral single–pass transmembrane protein and can be formatted as a secreted form

**Fig 2:**
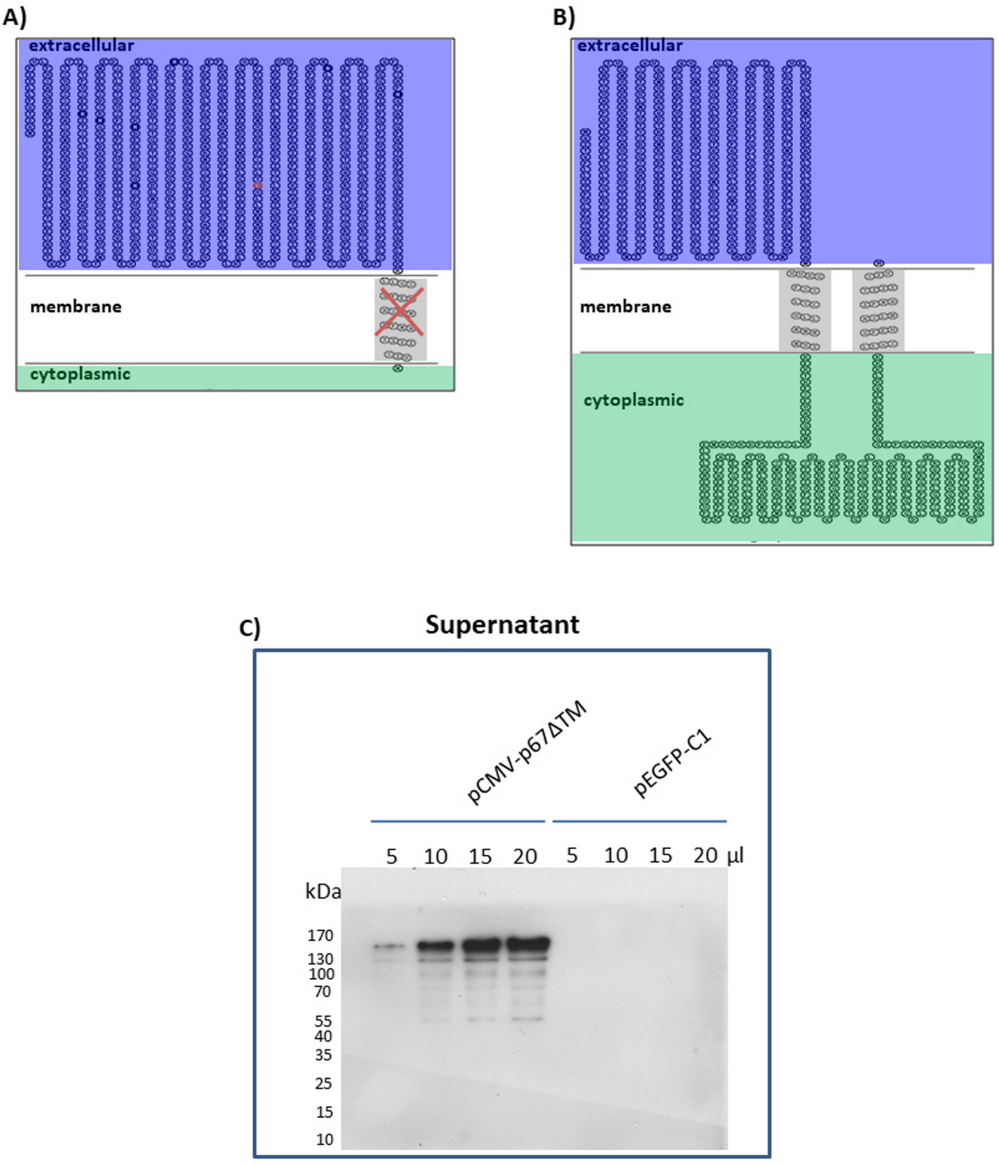
p67 topology prediction and secretion. Possible p67 topological predictions based on its hydrophobic regions. **A)** As single pass transmembrane protein and **B)** as double pass transmembrane protein. **C)** Western immunoblotting of pCMV-p67ΔTM (p67secreted form) transfected HEK 293T cells supernatant. Lanes were loaded with different amounts of clarified cell serum free medium supernatant at 48 hours post transfection (5, 10, 15 and 20 μL, corresponding to 0.05 μg, 0.1 μg, 0.15 μg and 0.2 μg respectively); cells supernatant from pEGFPC-1 transfected cells served as negative controls (*Mock*).

The presence of two hydrophobic regions, one corresponding to the putative transmembrane domain (from aa686 to aa708) and the other from aa407 to aa425, could give rise to two different potential topologies **(Fig 2A** and **Fig 2B)**. Therefore to address this, it was assumed that the removal of the putative transmembrane domain would allow p67 to be secreted, giving credit to the protein topology designed in **Fig 2A**. A mammalian expression vector, pCMV-p67ΔTM, with the transmembrane domain deleted from the p67 ORF coding sequence was constructed. When HEK 293T cells were transfected with pCMV-p67ΔTM, p67 was secreted in the cell culture supernatant **(Fig 2C)** and reaching a concentration of ~10 μg/mL after 48 hours of incubation with serum free medium, thus confirming the presence of a single transmembrane domain and categorizing p67 as a *Type I integral single–pass transmembrane protein* when expressed in mammalian cells.

### p67 expressed in mammalian cells migrates in SDS-PAGE with a higher molecular weight than expected and is not glycosylated

**Fig 3:**
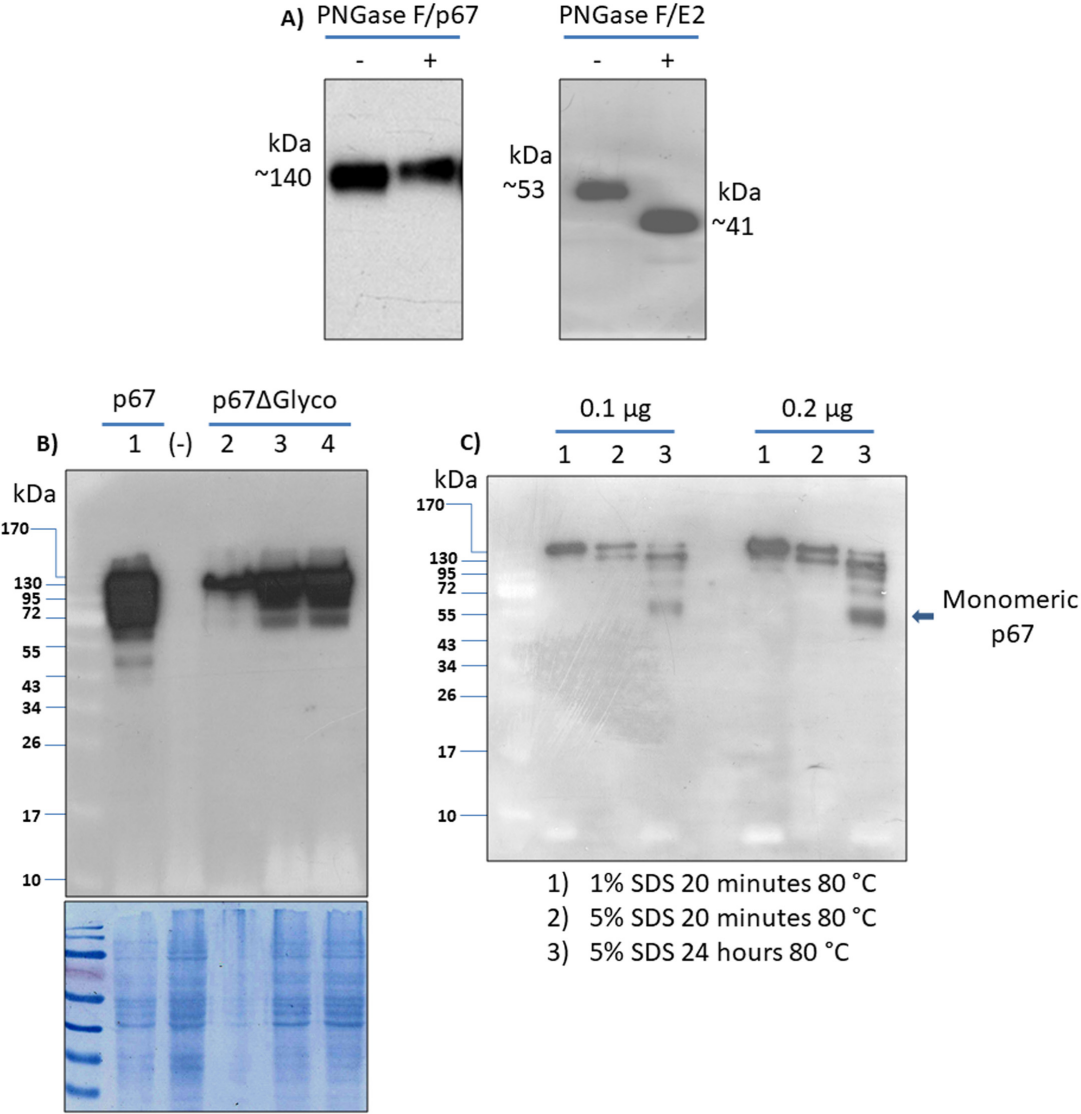
p67 analysis. **A)** Western immunoblotting of p67 secreted protein (~0.1 μg/lane corresponding to 10 μL of pCMV-p67ΔTM transfected HEK 293T serum free medium supernatant) treated (+) or untreated (-) with PNGase F. A positive control for PNGase F activity was established with a similar amount of secreted BVDV glycoprotein E2, whose molecular weight moved from 53 to 41 kDa. **B)** Western immunoblotting of the native p67 protein (pCMV-p67) compared with different amount of mutated p67 (pCMV-p67ΔGlyco), showing the same electrophoretic mobility. Lane 1 corresponds to native p67 (20 μg/lane of total protein extract). Lanes 2, 3 and 4 correspond to different protein amounts (15, 30 and 45 μg/lane respectively of total protein extract) of mutated p67. Negative control (-) was established with pEGFP-C1 transfected cells extract (45 μg of total protein extract). **C)** Western immunoblotting of secreted p67 treated with different denaturating conditions.

Both the secreted form and the membrane linked form of mammalian expressed p67, when loaded in SDS-PAGE and detected by Western blotting, migrated with a lower mobility than expected **(Figs 1B and 2C).** The possibility of p67 expression in mammalian cells as a glycosylated protein was considered. Analysis of p67 using three different glycosylation site prediction programs (Glyco EP, http://www.imtech.res.in/cgibin/glycoep/glyechk?job=932&tim=45; NetGlyc 1.0, http://www.cbs.dtu.dk/services/NetNGlyc/; Protter, http://wlab.ethz.ch/protter/#) yielded highly concordant results: all of the programs predicted the same seven putative N-linked glycosylation sites **(S2 Fig)** but no O-linked glycosylation sites. To validate this *in silico* prediction, pCMV-p67ΔTM transfected HEK 293T cells secreting p67 were treated with the glycosidases PNGase F **(Fig 3A)** but failed to reduce the molecular size of p67 when compared with undigested control p67. In order to eliminate any doubt regarding the glycosylation status of p67, a mutated form of p67, in which the seven potentially glycosylated asparagine residues were substituted with glutamine **(S3 Fig)** was constructed by gene synthesis pCMV-p67ΔGlyco and expressed in mammalian cells. Again, no reduction in molecular size between the mutated and non-mutated p67 was observed by Western immunoblotting **(Fig 3B)**. This further corroborates the fact that p67 expressed by mammalian cells is not glycosylated. In light of these data, a possible state of p67 aggregation, indestructible by normal denaturing conditions of an SDS-PAGE, was hypothesized. In support of this hypothesis, by increasing the SDS-PAGE loading buffer denaturing conditions with higher SDS concentration (5%) and extending heat treatment length up to 24 hours at 80°C, it was possible to shift the p67 molecular size to ~67 kDa **(Fig 3C)**.

### Vectored p67 is expressed by transduced mammalian cells and maintains immunogenic properties

**Fig 4:**
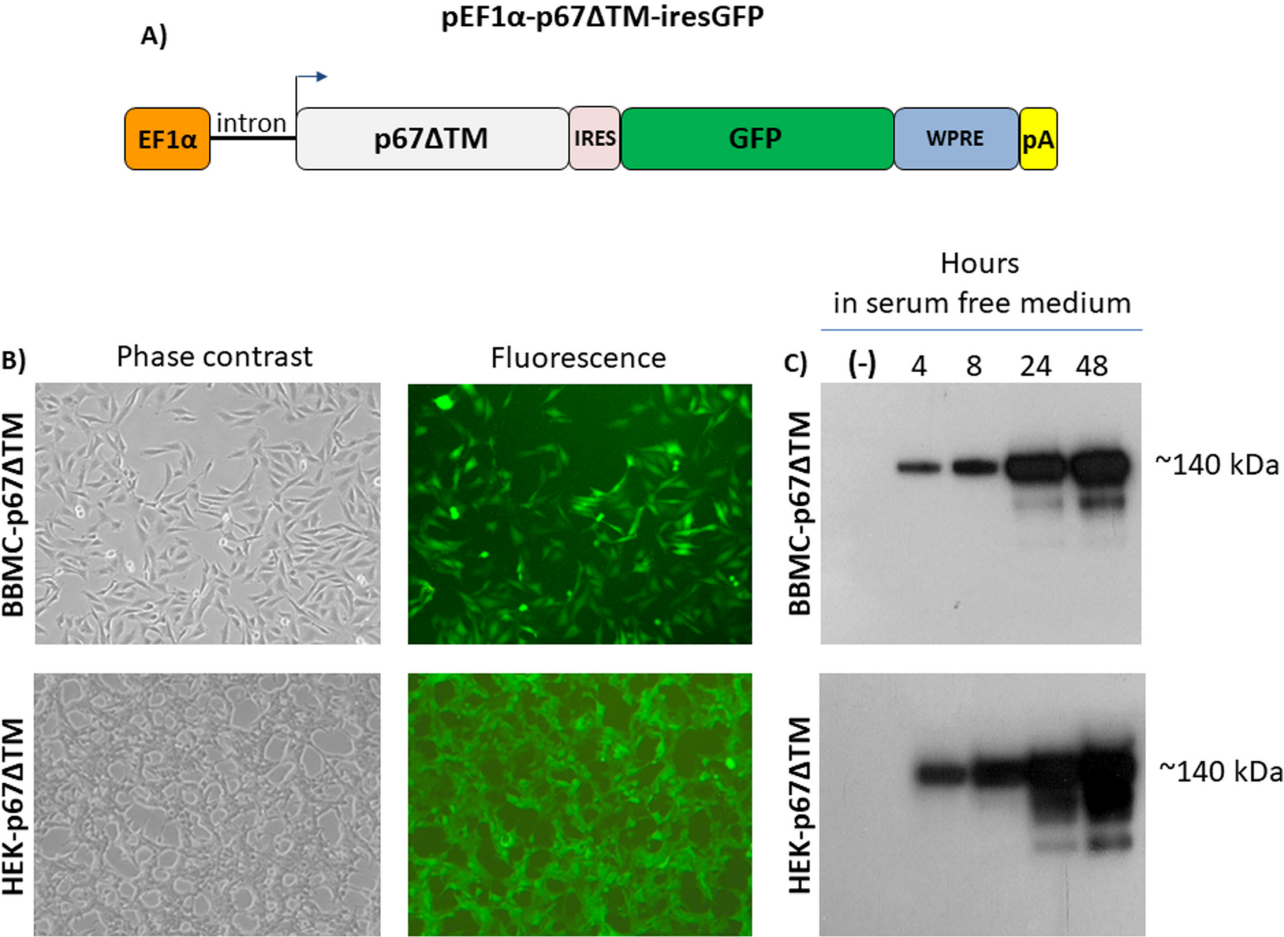
Generation of stably secreted p67 expressing cells. **A)** Diagram not on scale of pEF1α-p67ΔTM-iresGFP lentiviral transfer vector. **B)** Representative images (10X) of pEF1α-p67ΔTM-iresGFP transduced BBMC and HEK cells. **C)** Western immunoblotting of serum free medium at different times post collection, coming from BBMC-p67ΔTM and HEK-p67ΔTM cultures. Stably transduced cells were grown in presence of FBS and when reached confluence the medium was substituted with serum free medium. Protein secretion was tested by Western immunoblotting at 4, 8, 24 and 48 hours post medium change. In each line 15 μL of conditioned serum free medium was loaded. The negative control (-) was established with a similar lentiviral vector delivering only GFP (pEF1α-iresGFP). BBMC-p67ΔTM produced ~5μg/mL at 48 hours, whereas HEK-p67ΔTM ~7μg/mL.

**Fig 5:**
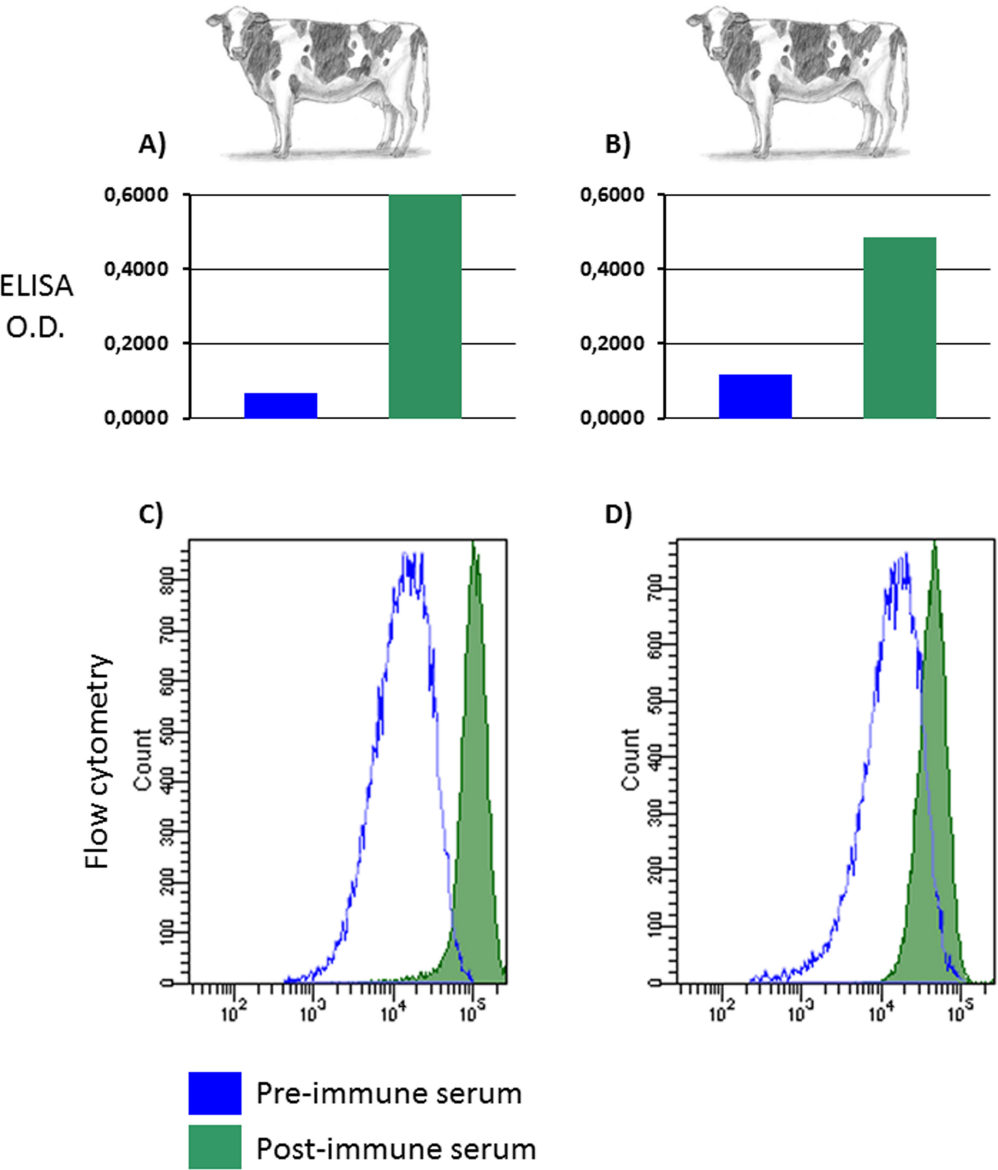
p67 immunogenic properties. Diluted sera (1/100) from two **(A and B)** inoculated cattle, were compared with pre-immune sera for anti-p67 antibodies by ELISA. Antibodies detected were expressed as the optical density at 450 nm. The same sera were test by flow cytometry on pCMV-p67 transfected cells **(C and D respectively)**. The green peak correspond to the post-immune sera from p67 immunized cattle and the blue peak corresponds to the pre-immune sera of the same animals.

A lentiviral transfer vector, pEF1α-p67ΔTM-iresGFP, delivering the secreted form of p67, under the transcriptional control of the elongation factor 1 alpha promoter (EF1α), followed by an internal ribosomal entry site (IRES), the GFP ORF and the woodchuck hepatitis virus post transcriptional regulatory element, was constructed **(Fig 4A)**. After the reconstitution of replication incompetent lentiviral particles, BBMC and HEK 293T cells were transduced and cell lines constitutively secreting p67 antigen into the medium supernatant were obtained (BBMC-p67ΔTM and HEK-p67ΔTM) **(Fig 4B and 4C)**. These cell lines represent a p67 antigen source to be employed for different purposes, such as immunization studies and as a diagnostic tool for anti-p67 antibody detection. In fact, p67 antigen collected from HEK-p67ΔTM serum free medium supernatant and then adjuvanted, was successfully employed to generate an anti-p67 antibody response in cattle, as detected by ELISA **(Fig 5A and 5B)** and flow cytometry **(Fig 5C and 5D)**.

Different pEF1α-p67ΔTM-iresGFP transduced cell lines (Goat Skin Stromal cells (GSSC), Swine Adipose Derived Stromal cells (SADSC), Equine Adipose Derived Stromal cells (EADSC) and Alpaca Skin Stromal cells (ASSC)) originated from different animal species were further generated and all of them expressed p67 protein in stable and soluble form **(S4 Fig)**.

## Discussion

Recent developments in artificial gene synthesis have enabled synthetic genes construction. De novo gene synthesis is a valuable synthetic biological tool for biotechnological studies which typically aim to improve tolerance to toxic molecules, retrofit existing biosynthetic pathways, design novel biosynthetic pathways and/or enhance heterologous protein production [32,33]. In the field of recombinant protein production, natural genes found in wild-type organisms are usually transformed into heterologous hosts for recombinant expression. This approach typically results in poorly expressed recombinant proteins since the wild-type foreign genes have not evolved for optimum expression in the host. Thus, it is highly desirable to harness the flexibility of synthetic biology to create customized artificial gene designs, optimal for heterologous protein expression. The degeneracy of the genetic code leads to a situation whereby most of the amino acids can be encoded by two to six synonymous codons. The synonymous codons are not equally utilized to encode the amino acids, thus resulting in phenomenon of codon usage bias. Importantly, codon usage bias has been shown to correlate with gene expression level, and it has been proposed as an important design parameter for enhancing recombinant protein production in heterologous host expression [34–36]. Based on this information, the popular web-based software, known as the Java Codon Adaptation Tool (JCat), allowed us to customize the *T*. *parva* p67 ORF for its expression in mammalian cells. Although we did not compare the adapted p67 ORF with the un-adapted one in terms of expression efficiency, the adapted p67 was expressed by mammalian cells both as membrane-linked and secreted form. Of note, the p67 ORF codon usage adaptation increased its GC content from 43 to 68%, which likely also influenced expression efficiency. Previous studies have shown that GC-rich genes in mammalian cells can be expressed 100-fold more efficiently than their GC-poor counterpart due to increased steady-state mRNA levels [37].

Based on its amino acids composition, p67 has a predicted molecular weight of ~67 kDa; however its migration through the denaturant SDS-PAGE was slower than predicted, at roughly twice the expected size, for both the membrane-linked and the secreted form. *In silico* analyses of the p67 amino acid composition using different softwares identified several N-linked glycosylation sites which could explain the unexpected electrophoretic migration. To test this hypothesis, the p67 supernatant was digested with PNGase F, and the p67 ORF was mutated by substituting glutamine for asparagine in the putative N-glycosylation sites. Glutamine was chosen because of its close structural similarity to asparagine, but found out to be unable of being linked to N-acetylglucosamine or fucose. However, none of these manipulations prevented the unexpected migration of p67 in SDS-PAGE.

A lower SDS-PAGE mobility of p67 was previously described by other researchers [28,38,39], during the attempt to express p67 as a full length in *E*. *coli*. It is very well known that *E*. *coli* is not able to glycosylate proteins, or at least recombinant proteins. The authors proposed that this anomalous mobility was due to the high serine/threonine (28%) and glutamate/glutamine (18%) protein composition [28,38,39]. When these observations were combined, glycosylation of p67 expressed by mammalian cells was excluded. In Western blots of the p67 membrane linked form **(Fig 1B),** two abundant bands, one corresponding to the p67 expected size and another one of larger size, were present, whereas in Western blots of the p67 secreted form, only the band with lower mobility was present **(Fig 2C)**. Based on these observations, it was reasoned that at some stage during p67 translation, membrane sorting and secretion, the protein could form small soluble dimeric aggregates. Likely, this could happen inside the Golgi apparatus when the protein, after translation, is accumulated within the Golgi vesicles and reaches high local concentration that could allow its aggregation. This might explain why the secreted form is represented by a single band with low mobility. To investigate its presumed state of aggregation, p67 was subjected to more drastic denaturing conditions which returned the mobility to ~67 kDa.

Since cells transiently transfected with a plasmid delivering p67ΔTM expression cassette allowed us to collect p67 protein in the transfected cell medium, it was of interest to generate a cell line constitutively and stably secreting p67 to be employed for different purposes. Therefore, a third-generation, replication-incompetent lentiviral vector delivering p67ΔTM expression cassette was constructed and HEK 293T cells were successfully transduced with an efficiency close to 100% as measured by GFP expression. The GFP ORF was in a bicistronic form with p67ΔTM ORF by an IRES sequence [40,41], thus the level of GFP expression in the transduced cells should reflect the expression level of the ORF upstream to the IRES which, in this specific case, is p67. Although this was not investigated in this work, because the pool of transduced cells produced enough p67 in the medium of HEK-p67ΔTM, the amount of p67 could be strongly increased by simply sorting HEK-p67ΔTM cells with the highest GFP expression. Moreover, secreted p67 was highly soluble and purification was not needed since HEK-p67ΔTM could be maintained in serum-free medium, allowing the collection of supernatant almost free of nonspecific protein. This is a great advantage when a secreted protein needs to be used as an antigen for immunization purposes or for diagnosis. In fact, serum-free medium supernatant coming from HEK-p67ΔTM cells was successfully employed to immunize goats and cattle. The pEF1α-p67ΔTM-iresGFP lentiviral vector was used to generate many other cell lines stably expressing p67, coming from different animal species, including the bovine, which is *T parva* natural host. These cell lines can be used as p67 antigen cargos for cell-based immunization.

In the present piece of work the production of *T parva* p67 antigen, either as a membrane linked or as a secreted form was successfully achieved. The general work-flow we proposed here could be applied for the production of other Apicomplexan antigens to be delivered by mammalian expression vectors such as viral vectors, plasmid vector injection or gene gun, cell based immunization or simply as secreted antigens produced in mammalian cells.

## Supporting information

S1 Fig Human codon usage adapted and un-adapted sequence. Human codon usage undapted **(A)** and adapted **(B)** p67 sequence and their GC content respectively **(C and D)**.
(PDF)

S2 Fig Representative p67 N-linked glycosylation sites. (PDF)

S3 Fig p67 mutation. **A)** Native and **B)** mutated p67 protein sequences, indicating the Asparagines residues (N; green) in the native p67 and substituted with Glutamine (Q, green) in the mutated p67, along with the molecular structure of the two amino acids which differ only to a carbon atom.
(PDF)

S4 Fig p67 expression in transduced cell lines. (PDF)
