# Supplementary material for: Assessment and optimization of Theileria parva sporozoite full-length p67 antigen expression in mammalian cells
Source: PLoS Negl Trop Dis. 2017 Aug 11;11(8):e0005803. doi: 10.1371/journal.pntd.0005803 (PMC5568440; doi:10.1371/journal.pntd.0005803)
Supplement: S1 Fig — Human codon usage undapted (A) and adapted (B) p67 sequence and their GC content respectively (C and D). (PDF) [file pntd.0005803.s001.pdf]

A)

```
ATGCAAATAACTCAGTTTTTGCTGATCATTCCGGTCTTATTTGTATCAGC      50
AGGGGACAAAATGCCTACGGAGGAACAACCATTTCCTTCTAGGCTTGGTC      100
CCCTAGTAACCTTGGAATCAGCCATAACACAACCTACCGCCGTGTACACA      150
ATGAGGACAGTTGGTAATGTGGCAAAGGCAGCAAAGGCATGGAAGTCAGC      200
AGTATCATCTTCAGATGTCTCTACCACTATTCCCACTCCAGTTTCGGAAG      250
AAAATATCACATCAACTCTTCAAACACAAACGGAAGAAGTTCCTGCTGCA      300
AGCGGCTCAGATTCATACACTGTAACAAATTTGGTACAAACACAATCCCA      350
AGTTCAGGATAATGTAAAGCAACAGCAAGATACTAAGGGGAACAGATCAG      400
ATTCCGAAGAAGAAAATGAAGATAGCACCCTTAGTACAGATGTCTCTCCG      450
ACCATTCCTACTCCAGTTTCGGAAGAAATTATCACACCTACTCTTCAAGC      500
ACAAACGAAAGAAGAAGTTCCTCCTGCAGACCTCTCAGATCAAGTTCCGT      550
CAAACGGCTCAGACTCCGAAGAAGAAGATAATAAATCCACCTCATCTAAA      600
GATGAAAAGGAACTCAAAAAAACTCTACAACCCGGAAAAACATCCACAGG      650
TGAAACTACATCGGGCCAAGATCTTAATTCAAAACAACAGCAAACTGGTG      700
TATCAGATCTAGCCAGTGGATCACACTCTTCTGGACTTAAAGTACCTGGA      750
GTAGGAGTTCCAGGTGCAGTTTCTCCCCAAGGAGGTCAATCTTTAGCTTC      800
GAATACATCTAGAGAAGGTCAGGCGCAGCATCAACAGGTAAGAGATGGAG      850
ATGGTAGAGTTATTGAGCCTAAAATTGGATTACCCGGACCTCCATCTGCG      900
CCAGTACCATCACCAGGAGCGCCCGGAATAATTGTTAGAGAATCAGGCAA      950
TAGGGCAATGGATATTGTACAGTTTTTAGGAAGATTTAAACCAGAACCAA      1000
GGGCATATGAAGGGGAAAGAACAAATGTAGCAGAACTAAAAAAATTCCTA      1050
TTTGAAGAACTTGAATCTTTGGTAAACACTCTAATAGAATTGAAATTAGC      1100
AATTGCAAGCGACTTTGTTGAAATCACTGATGGTTTGAGAAAGAATACTA      1150
AAGATCATGAAGCCAGATTGAAGTTGCTAAGAGGTGTAGAATTCACTAAG      1200
AGGAAAAGTGTCGCCAACGTAGTAAAGGGATTCAGTTCTTTGTACTGTGT      1250
GCTTTTAATGAATATGAACGTCATCAAAGAAAAAACGAAAGAATCTGAAG      1300
TAGCAGATGGCATTTGGAAACTGTCTACAATCCCAGATAAAGTAGCAAAT      1350
GAACTTTTGTTAGCTATGGAAAAGATCGTGGTCCCACCAAAAACCCCTGA      1400
ACTAGAAGAAGCGTTTGAGGCAATCGAGTTTGGTTTCAAAATAGCATACT      1450
ACGCAACCAAAGACATCCTCTCAAGTATAGAAAACACAGTTCACAACTTG      1500
ATGCACGCCAAAAATTATGAAGAGAATTTTATTGCTCAAGTAAGAAACTC      1550
TCTAAGGATGGTACCACACCAGATGAACTTGACTGAATCGTCGTTTGTAA      1600
TTAAAATCTCAGACATGATGCGCAGAAGAGGAACAGCTAGTCAGGACGAA      1650
CCAGCAGGAGCTGGGTCCGGAGTAACACCAGGACGAGGATCATCAGGTAC      1700
GGGACGAGCAGCAGGAACGGGAGGGGGATCACTGAGGGGATTAGACTTAA      1750
GTGAAGAAGAAGTTAAGAAAATCTTGGATGAAATAGTGAAAGATCCCAGC      1800
GACGGAGAACTTGGACTCGGAGACTTAAGTGACCCAAGTGGAAGATCATC      1850
CGAAAGACAACCCTCACTCGGACCTTCACTTGTAATAACTGATGGACAAG      1900
CAGGACCCACAATAGTATCTCCAACAGGGCCCACAATAGCAGCTGGAGGA      1950
GAACAACCACCTTCAGCTCCTAATGGAACCGCAACGGGGCCAGCAGGAAC      2000
ACAACCTGAGGGAGGAGAGAAGAAAGAAGGATTGATACAGAAGCTCAAGA      2050
AAAAACTCCTGGGGTCTGGATTCGAAGTCGCGAGTCTTATGATACCAATG      2100
GCGACAATAATTATCAGCATCGTCCACTAA
```

B)

```
ATGCAGATCACCCAGTTCCTGCTGATCATCCCCGTGCTGTTCGTGAGCGC      50
CGGCGACAAGATGCCCACCGAGGAGCAGCCCTTCCCCAGCCGCCTGGGCC      100
CCCTGGTGACCCTGGAGAGCGCCATCACCCAGCCCACCGCCGTGTACACC      150
ATGCGCACCGTGGGCAACGTGGCCAAGGCCGCCAAGGCCTGGAAGAGCGC      200
CGTGAGCAGCAGCGACGTGAGCACCACCATCCCCACCCCCGTGAGCGAGG      250
AGAACATCACCAGCACCCTGCAGACCCAGACCGAGGAGGTGCCCGCCGCC      300
AGCGGCAGCGACAGCTACACCGTGACCAACCTGGTGCAGACCCAGAGCCA      350
GGTGCAGGACAACGTGAAGCAGCAGCAGGACACCAAGGGCAACCGCAGCG      400
ACAGCGAGGAGGAGAACGAGGACAGCACCCTGAGCACCGACGTGAGCCCC      450
ACCATCCCCACCCCCGTGAGCGAGGAGATCATCACCCCCACCCTGCAGGC      500
CCAGACCAAGGAGGAGGTGCCCCCCGCCGACCTGAGCGACCAGGTGCCCA      550
GCAACGGCAGCGACAGCGAGGAGGAGGACAACAAGAGCACCAGCAGCAAG      600
GACGAGAAGGAGCTGAAGAAGACCCTGCAGCCCGGCAAGACCAGCACCGG      650
CGAGACCACCAGCGGCCAGGACCTGAACAGCAAGCAGCAGCAGACCGGCG      700
TGAGCGACCTGGCCAGCGGCAGCCACAGCAGCGGCCTGAAGGTGCCCGGC      750
GTGGGCGTGCCCGGCGCCGTGAGCCCCCAGGGCGGCCAGAGCCTGGCCAG      800
CAACACCAGCCGCGAGGGCCAGGCCCAGCACCAGCAGGTGCGCGACGGCG      850
ACGGCCGCGTGATCGAGCCCAAGATCGGCCTGCCCGGCCCCCCCAGCGCC      900
CCCGTGCCCAGCCCCGGCGCCCCCGGCATCATCGTGCGCGAGAGCGGCAA      950
CCGCGCCATGGACATCGTGCAGTTCCTGGGCCGCTTCAAGCCCGAGCCCC      1000
GCGCCTACGAGGGCGAGCGCACCAACGTGGCCGAGCTGAAGAAGTTCCTG      1050
TTCGAGGAGCTGGAGAGCCTGGTGAACACCCTGATCGAGCTGAAGCTGGC      1100
CATCGCCAGCGACTTCGTGGAGATCACCGACGGCCTGCGCAAGAACACCA      1150
AGGACCACGAGGCCCGCCTGAAGCTGCTGCGCGGCGTGGAGTTCACCAAG      1200
CGCAAGAGCGTGGCCAACGTGGTGAAGGGCTTCAGCAGCCTGTACTGCGT      1250
GCTGCTGATGAACATGAACGTGATCAAGGAGAAGACCAAGGAGAGCGAGG      1300
TGGCCGACGGCATCTGGAAGCTGAGCACCATCCCCGACAAGGTGGCCAAC      1350
GAGCTGCTGCTGGCCATGGAGAAGATCGTGGTGCCCCCCAAGACCCCCGA      1400
GCTGGAGGAGGCCTTCGAGGCCATCGAGTTCGGCTTCAAGATCGCCTACT      1450
ACGCCACCAAGGACATCCTGAGCAGCATCGAGAACACCGTGCACAACCTG      1500
ATGCACGCCAAGAACTACGAGGAGAACTTCATCGCCCAGGTGCGCAACAG      1550
CCTGCGCATGGTGCCCCACCAGATGAACCTGACCGAGAGCAGCTTCGTGA      1600
TCAAGATCAGCGACATGATGCGCCGCCGCGGCACCGCCAGCCAGGACGAG      1650
CCCGCCGGCGCCGGCAGCGGCGTGACCCCCGGCCGCGGCAGCAGCGGCAC      1700
CGGCCGCGCCGCCGGCACCGGCGGCGGCAGCCTGCGCGGCCTGGACCTGA      1750
GCGAGGAGGAGGTGAAGAAGATCCTGGACGAGATCGTGAAGGACCCCAGC      1800
GACGGCGAGCTGGGCCTGGGCGACCTGAGCGACCCCAGCGGCCGCAGCAG      1850
CGAGCGCCAGCCCAGCCTGGGCCCCAGCCTGGTGATCACCGACGGCCAGG      1900
CCGGCCCCACCATCGTGAGCCCCACCGGCCCCACCATCGCCGCCGGCGGC      1950
GAGCAGCCCCCCAGCGCCCCCAACGGCACCGCCACCGGCCCCGCCGGCAC      2000
CCAGCCCGAGGGCGGCGAGAAGAAGGAGGGCCTGATCCAGAAGCTGAAGA      2050
AGAAGCTGCTGGGCAGCGGCTTCGAGGTGGCCAGCCTGATGATCCCCATG      2100
GCCACCATCATCATCAGCATCGTGCACTAA
```

C)

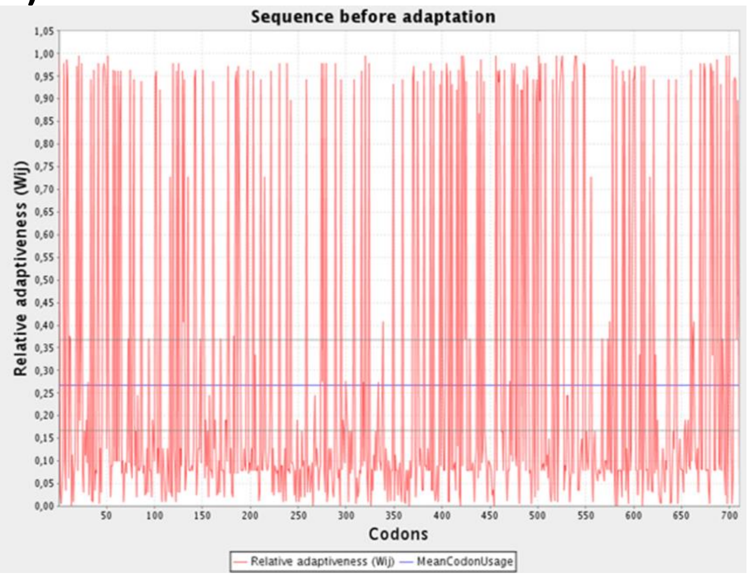

GC-content of the original sequence (43%)

D)

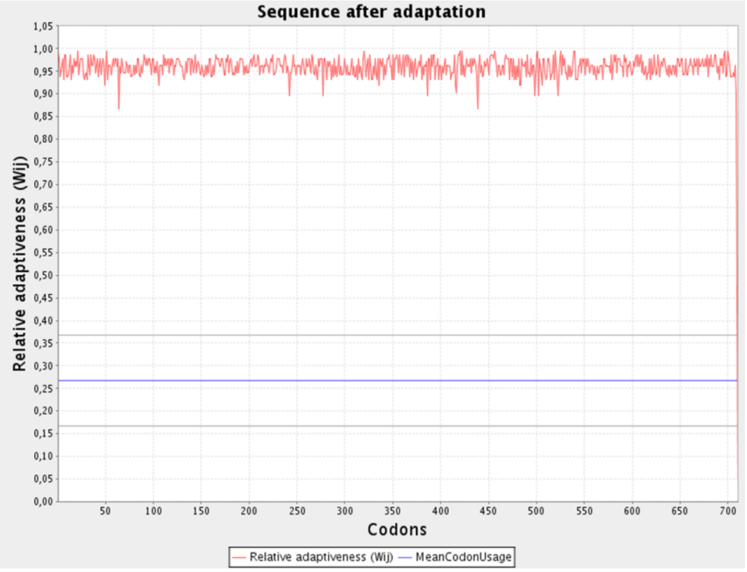

GC-content of the adapted sequence (68%)

Supplementary Figure 1) Human codon usage un-adapted **(A)** and adapted **(B)** p67 sequence and their GC content respectively **(C and D)**.
