# Supplementary material for: Assessment and optimization of Theileria parva sporozoite full-length p67 antigen expression in mammalian cells
Source: PLoS Negl Trop Dis. 2017 Aug 11;11(8):e0005803. doi: 10.1371/journal.pntd.0005803 (PMC5568440; doi:10.1371/journal.pntd.0005803)
Supplement: S2 Fig — (PDF) [file pntd.0005803.s002.pdf]

```
MQITQFLLIIPVLFVSAGDKMPTEEQPFPSRLGPLVTLESAITQPTAVYTMRTVGNVAKAAKAWKSAVSSSDVSTTIPTP
80
VSEENITSTLQTQTEEVPAASGSDSYTVTNLVQTQSQVQDNVKQQQDTKGNRSDSEEENEDSTLSTDVSPTIPTPVSEEI
160
ITPTLQAQTKEEVPPADLSDQVPSNGSDSEEEDNKSTSSKDEKELKKTLQPGKTSTGETTSGQDLNSKQQQTGVSDLASG
240
SHSSGLKVPGVGVPGAVSPQGGQSLASNTSREGQAQHQQVRDGDGRVIEPKIGLPGPPSAPVPSPGAPGIIVRESGNRAM
320
DIVQFLGRFKPEPRAYEGERTNVAELKKFLFEELESLVNTLIELKLAIASDFVEITDGLRKNTKDHEARLKLLRGVEFTK
400
RKSVANVVKGFSSLYCVLLMNMNVIKEKTKESEVADGIWKLSTIPDKVANELLLAMEKIVVPPKTPELEEAFEAIEFGFK
480
IAYYATKDILSSIENTVHNLMHAKNYEENFIAQVRNSLRMVPHQMNLTESSFVIKISDMMRRRGTASQDEPAGAGSGVTP
560
GRGSSGTGRAAGTGGGSLRGLDLSEEEVKKILDEIVKDPSDGELGLGDLSDPSGRSSERQPSLGPSLVITDGQAGPTIVS
640
PTGPTIAAGGEQPPSAPNGTATGPAGTQPEGGEKKEGLIQKLKKKLLGSGFEVASLMIPMATIIISIVH
................................................................................
80
....N.............................................N.............................
160
........................N........N..............................................
240
...........................N....................................................
320
................................................................................
400
................................................................................
480
.............................................N..................................
560
................................................................................
640
.................N...................................................
720

(Threshold=0.5)
```

SeqName	Position		Potential	Jury agreement	N-Glyc result
Sequence	85	NITS	0.6992	(8/9)	+
Sequence	131	NRSD	0.5946	(8/9)	+
Sequence	185	NGSD	0.6900	(9/9)	++
Sequence	194	NKST	0.5820	(7/9)	+
Sequence	268	NTSR	0.6198	(7/9)	+
Sequence	526	NLTE	0.6267	(8/9)	+
Sequence	658	NGTA	0.7284	(9/9)	++

Supplementary Figure 2. Representative p67 N-linked glycosylation sites. Potentially glycosylated Asparagines (N) are in red and are flanked by consensus amino acids in blue. The value crossing the default threshold of 0.5, represents a predicted glycosylated site (as long as it occurs in the required sequon Asn-Xaa-Ser/Thr without Proline at Xaa). The 'potential' score is the averaged output of nine networks. For further information, the jury agreement column indicates how many of the nine networks support the prediction.
