# Supplementary material for: Assessment and optimization of Theileria parva sporozoite full-length p67 antigen expression in mammalian cells
Source: PLoS Negl Trop Dis. 2017 Aug 11;11(8):e0005803. doi: 10.1371/journal.pntd.0005803 (PMC5568440; doi:10.1371/journal.pntd.0005803)
Supplement: S3 Fig — A) Native and B) mutated p67 protein sequences, indicating the Asparagines residues (N; green) in the native p67 and substituted with Glutamine (Q, green) in the mutated p67, along with the molecular structure of the two amino acids which differ only to a carbon atom. (PDF) [file pntd.0005803.s003.pdf]

A)

```
MQITQFLLIIPVLFVSAGDKMPTEEQPFPSRLGPLVTLESAITQPTAVYTMRTVGNVAKA
AKAWKSAVSSSDVSTTIPTPVSEENITSTLQTQTEEVPAASGSDSYTVTNLVQTQSQVQD
NVKQQQDTKGNRSDSEEENEDSTLSTDVSPTIPTPVSEEIITPTLQAQTKEEVPPADLSD
QVPSNGSDSEEEDNKSTSSKDEKELKKTLQPGKTSTGETTSGQDLNSKQQQTGVSDLASG
SHSSGLKVPGVGVPGAVSPQGGQSLASNTSREGQAQHQQVRDGDGRVIEPKIGLPGPPSA
PVPSPGAPGIIVRESGNRAMDIVQFLGRFKPEPRAYEGERTNVAELKKFLFEELESLVNT
LIELKLAIASDFVEITDGLRKNTKDHEARLKLLRGVEFTKRKSVANVVKGFSSLYCVLLM
NMNVIKEKTKESEVADGIWKLSTIPDKVANELLLAMEKIVVPPKTPELEEAFEAIEFGFK
IAYYATKDILSSIENTVHNLMHAKNYEENFIAQVRNSLRMVPHQMNLTESSFVIKISDMM
RRRGTASQDEPAGAGSGVTPGRGSSGTGRAAGTGGGSLRGLDLSEEEVKKILDEIVKDPS
DGELGLGDLSDPSGRSSERQPSLGPSLVITDGQAGPTIVSPTGPTIAAGGEQPPSAPNGT
ATGPAGTQPEGGEKKEGLIQKLKKKLLGSGFEVASLMIPMATIIISIVH-
```

Asparagine

N

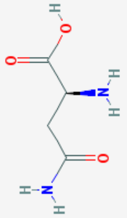

B)

```
MQITQFLLIIPVLFVSAGDKMPTEEQPFPSRLGPLVTLESAITQPTAVYTMRTVGNVAKA
AKAWKSAVSSSDVSTTIPTPVSEEQITSTLQTQTEEVPAASGSDSYTVTNLVQTQSQVQD
NVKQQQDTKGQRSDSEEENEDSTLSTDVSPTIPTPVSEEIITPTLQAQTKEEVPPADLSD
QVPSQGSDSEEEDQKSTSSKDEKELKKTLQPGKTSTGETTSGQDLNSKQQQTGVSDLASG
SHSSGLKVPGVGVPGAVSPQGGQSLASQTSREGQAQHQQVRDGDGRVIEPKIGLPGPPSA
PVPSPGAPGIIVRESGNRAMDIVQFLGRFKPEPRAYEGERTNVAELKKFLFEELESLVNT
LIELKLAIASDFVEITDGLRKNTKDHEARLKLLRGVEFTKRKSVANVVKGFSSLYCVLLM
NMNVIKEKTKESEVADGIWKLSTIPDKVANELLLAMEKIVVPPKTPELEEAFEAIEFGFK
IAYYATKDILSSIENTVHNLMHAKNYEENFIAQVRNSLRMVPHQMQLTESSFVIKISDMM
RRRGTASQDEPAGAGSGVTPGRGSSGTGRAAGTGGGSLRGLDLSEEEVKKILDEIVKDPS
DGELGLGDLSDPSGRSSERQPSLGPSLVITDGQAGPTIVSPTGPTIAAGGEQPPSAPQGT
ATGPAGTQPEGGEKKEGLIQKLKKKLLGSGFEVASLMIPMATIIISIVH-
```

Glutamine

Q

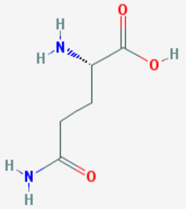

Supplementary Figure 3. p67 mutation. A) Native and **B)** mutated p67 protein sequences, indicating the Asparagines residues (N; green) in the native p67 and substituted with Glutamine (Q, green) in the mutated p67, along with the molecular structure of the two amino acids which differ only to a carbon atom.
