# Supplementary material for: Assessment and optimization of Theileria parva sporozoite full-length p67 antigen expression in mammalian cells
Source: PLoS Negl Trop Dis. 2017 Aug 11;11(8):e0005803. doi: 10.1371/journal.pntd.0005803 (PMC5568440; doi:10.1371/journal.pntd.0005803)
Supplement: S4 Fig — (PDF) [file pntd.0005803.s004.pdf]

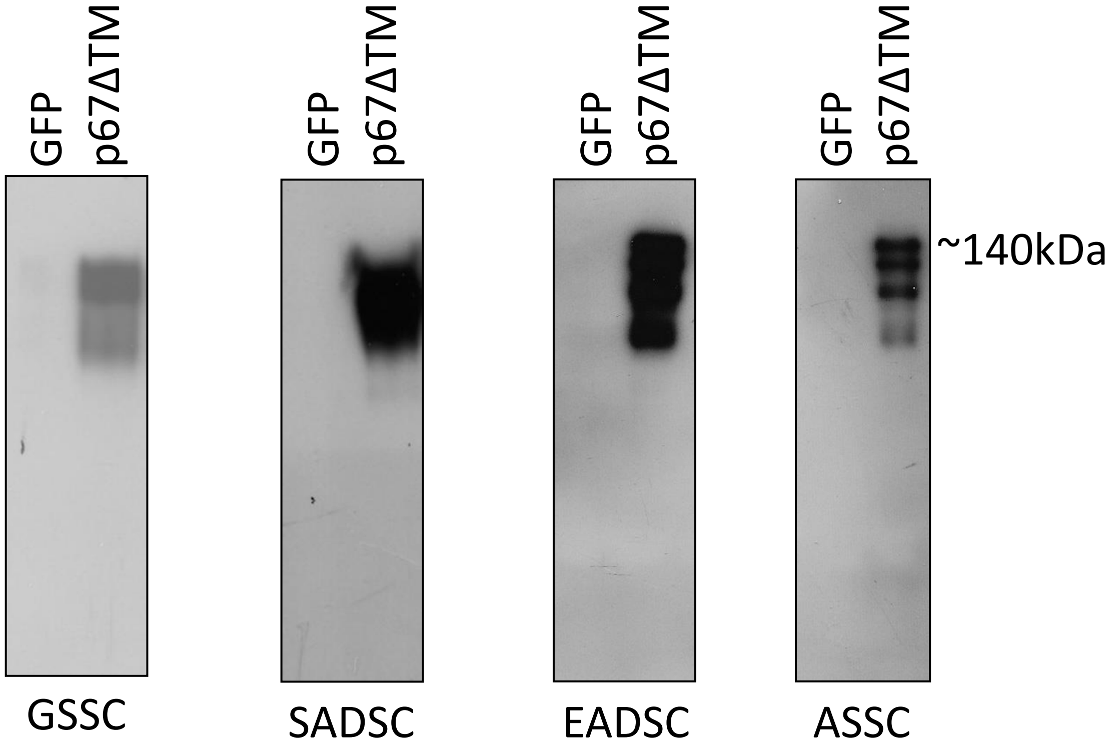

Western immunoblotting of serum free medium, coming from pEF1α-p67ΔTM-iresGFP transduced Goat Skin Stromal cells (GSSC), Swine Adipose Derived Stromal cells (SADSC), Equine Adipose Derived Stromal cells (EADSC) and Alpaca Skin Stromal cells (ASSC). Stably transduced cells were growth with the presence of FBS and when reached confluence the medium was substituted with serum free medium. Protein secretion was tested by Western immunoblotting at 48 hours post medium change. In each line 15 µl of conditioned serum free medium was loaded. The negative control (-) was established with a similar lentiviral vector delivering only GFP (EF1α-iresGFP).
